# Key Factors Analysis of Severity of Automobile to Two-Wheeler Traffic Accidents Based on Bayesian Network

**DOI:** 10.3390/ijerph19106013

**Published:** 2022-05-15

**Authors:** Lining Liu, Xiaofei Ye, Tao Wang, Xingchen Yan, Jun Chen, Bin Ran

**Affiliations:** 1 Faculty of Maritime and Transportation, Ningbo University, Fenghua Road 818#, Ningbo 315211, China; liulining1999@163.com; 2 School of Architecture and Transportation, Guilin University of Electronic Technology, Lingjinji Road 1#, Guilin 541004, China; 3 College of Automobile and Traffic Engineering, Nanjing Forestry University, Longpan Road 159#, Nanjing 210037, China; xingchenyan.acad@gmail.com; 4 School of Transportation, Southeast University, Si Pai Lou 2#, Nanjing 211189, China; chenjun@seu.edu.cn; 5 Department of Civil and Environmental Engineering, University of Wisconsin-Madison, Madison, WI 53706, USA; bran@seu.edu.cn

**Keywords:** big data and traffic safety, severity of accidents, automobile to two-wheeler traffic accidents, Kendall rank correlation, Bayesian network

## Abstract

The purpose of this paper is to analyze the complex coupling relationships among accident factors contributing to the automobile and two-wheeler traffic accidents by establishing the Bayesian network (BN) model of the severity of traffic accidents, so as to minimize the negative impact of automobile to two-wheeler traffic accidents. According to the attribution of primary responsibility, traffic accidents were divided to two categories: the automobile and two-wheeler traffic as the primary responsible party. Two BN accident severity analysis models for different primary responsible parties were proposed by innovatively combining the Kendall correlation analysis method with the BN model. A database of 1560 accidents involving an automobile and two-wheeler in Guilin, Guangxi province, were applied to calibrate the model parameters and validate the effectiveness of the models. The result shows that the BN models could reflect the real relationships among the influential factors of the two types of traffic accidents. For traffic accidents of automobiles and two-wheelers as the primary responsible party, respectively, the biggest influential factors leading to fatality were weather and visibility, and the corresponding fluctuations in the probability of occurrence were 32.20% and 27.23%, respectively. Moreover, based on multi-factor cross-over analysis, the most influential factors leading to fatality were: {Off-Peak Period → Driver of Two-Wheeler: The elderly → Driving Behavior of Two-Wheeler: Parking} and {Drunk Driving Two-Wheeler → Having a License of Automobiles → Visibility: 50 m~100 m}, respectively. The results provide a theoretical basis for reducing the severity of automobile to two-wheeler traffic accidents.

## 1. Introduction

As an important part of the transportation system, two-wheeler traffic plays an extremely important role in solving the short-distance trips by its virtue of light weight, convenient driving, and affordable characteristics. With the strengthening of people’s awareness of health and environmental protection, as well as the rapid development of sharing bikes and e-bikes, more and more people travel by two-wheeled vehicles [1]. Two-wheeled vehicles include bicycles, e-bikes, and motorcycles. By the end of 2019, there were about 90 million motorcycles, nearly 400 million bicycles, and 300 million e-bikes in China [2]. Meanwhile, e-bikes are growing at an annual rate of 20% [3]. The rapid growth of two-wheeled vehicles also causes serious safety problems. Traffic accidents related to two-wheeled traffic increase significantly every year, especially those with serious casualties. According to the annual report of traffic accidents in China, the total number of road traffic accidents in China was 247,646 in 2019, and 74,684 accidents related to two-wheeled traffic accounted for 30.15%, resulting in 100,888 casualties and RMB 170 million economic losses [2]. Automobile to two-wheeler traffic accidents are the main types of accidents related to two-wheeled traffic [4]. Therefore, it is necessary to conduct in-depth research on automobile to two-wheeler traffic accidents, explore their occurrence rules, and put forward effective preventive measures, which are of great significance for reducing the severity of automobile to two-wheeled traffic accidents and improving road traffic safety [5].

For a long time, the study of automobile to two-wheeler traffic accidents has shifted from the theory of collision mechanics to the analysis of various influential factors. Traffic accidents are mainly affected by vehicle behavior characteristics, road characteristics, and environment characteristics [6]. Because automobiles usually have a stronger structure than two-wheeled vehicles, their influences on traffic accidents are quite different [7]. Meanwhile, various factors and their own behavior also generate different influences on automobile and two-wheeler accidents [8]. Especially, when the primary responsible parties for traffic accidents are different, the main factors causing traffic accidents have significant differences. However, few existing studies took the classification of primary responsibilities leading to the accidents into account during traffic accident analysis. The Bayesian network (BN) model has been widely used in sample learning methods, network structure construction, and inference mechanism learning. Because of its powerful inference function and excellent result visualization capability, the BN model has gradually been used in the field of traffic accident analysis [9,10]. Therefore, this paper took the primary responsible party as the starting point to identify the key factors of automobile to two-wheeler traffic accidents and used the BN model to explore the coupling relationship between the key factors. We addressed the following research problems: (a) What are the key factors of traffic accidents in which automobiles or two-wheelers are the primary responsible party, respectively? (b) How should the road safety management strategy be formulated according to the influence mechanism learned from the results based on the BN model?

The traffic accidents with real datasets were divided to two categories: the automobile and two-wheeler traffic as the primary responsible party, respectively. Then, this paper identified the key factors of the traffic accident severity with the automobile the two-wheeler as the primary responsible party, respectively, and discussed the internal relationships among the different factors. Through the comprehensive comparison of these factors, the corresponding traffic control measures were put forward to improve the accident prevention system. Therefore, this paper established a correlation analysis method based on the Kendall rank correlation coefficient and the BN analysis model of accident severity. Firstly, the key influencing factors of the traffic accidents for the automobile and the two-wheeler as the primary responsible party were determined, respectively, by the Kendall rank correlation coefficient. Secondly, the BN model was established with the key factors as the nodes and the intrinsic correlation as the link. Thirdly, the sample data was trained by K2 algorithm based on the Calinski–Harabasz (CH) score. Finally, the conditional probability based on Bayesian estimation was used to verify the validity of the model.

## 2. Literature Review

Many scholars have researched automobile to two-wheeler traffic accidents. In the past, the main methods focused on applying the mechanical theory and experimental data to summarize empirical formulas, as well as using simulation data to reconstruct the accidents. For example, Deguchi et al. replaced the two-wheeler driver with the 50th percentile multi-rigid-body simulation dummy model in the MADYMO database and established the crash model with the multi-rigid-body dynamics simulation method to analyze the automobile–light two-wheeler collision accidents [11,12]. Husher et al. used momentum/energy, SMAC, and PC-crash methods to reconstruct a motorcycle accident and analyzed the influence of changes in parameters such as vehicle impact speed and impact point position on the simulation results [13].

Recently, the real data of automobile to two-wheeler traffic accidents was applied to study the causes of the accidents and put forward corresponding measures to reduce the number and loss of traffic accidents. Allen et al. conducted a questionnaire survey of injured drivers and a detailed inspection of the accident vehicles and locations. They summarized automobile to two-wheeler traffic accident data and used logistic regression analysis to determine that the driver’s misoperation was related to factors such as driver’s age, traffic flow, unreasonable driving speed, and road design [14]. Lin et al. designed orthogonal experiments to carry out weight analysis on the influential parameters of automobile to two-wheeler collision and used a logistic regression method to analyze real collision accidents [15,16]. Ahmad et al. analyzed moderate and severe Traumatic Brain Injury (TBI) cases caused by motorcycle crashes in Bandung and used a logistic regression model to determine the relationships between age, gender, alcohol, helmet use, and other factors and brain injury [17]. BoeleVos et al. analyzed the data on bicycle traffic accidents of Dutch cyclists aged 50 and above and determined the influence of the environmental factors on the bicycle accidents [18].

Although some studies considered the influence of the driving behaviors of both parties on the occurrence of the accident, they ignored the different influences of the primary and secondary responsibility for two parties on the automobile and two-wheeler accidents. In fact, the occurrence mechanism and key influential factors of traffic accidents in which the automobile is the primary responsible party are not exactly the same as those in which the two-wheeler is the primary responsible party. Furthermore, the differences in the behaviors are distinguished with the owners of responsibility for two parties during traffic accidents and create the different influence on the accident severity. Therefore, it is necessary to classify the primary responsibilities of two parties for the key factors analysis of severity of automobile to two-wheeler traffic accidents.

## 3. Data Description

### 3.1. Data Sources

The accident data were collected from the real traffic accident cases in Department of Transportation Guilin City, Guangxi Province, China. The data included 1560 accidents involving automobiles and two-wheelers in Guilin, Guangxi province, between January 2011 and December 2019.

### 3.2. Descriptive Analysis

Some of the original data are shown in Table 1. According to the injuries of the parties involved in the traffic accident, the accidents are classified into fatal accidents; severe accidents; minor accidents; and property damage. In particular, the criterion for classifying a severe accident and minor accident is whether the person involved in the accident loses the ability to act normally. The accident situation was statistically analyzed from five perspectives: accident characteristics, accident causes, accident liability, automobile information, and two-wheeler information. Data items in the dataset are recorded and analyzed by the Traffic Police department of Public Security Organ based on the real-world traffic accident cases. In particular, accident liability is determined as follows: After identifying and investigating the cause of traffic accidents, the Traffic Police determine the responsibility of the parties for the traffic accident according to the causal relationship between the parties’ violations and traffic accidents under the framework and provisions of Road Traffic Safety Law.

**Figure 1:**
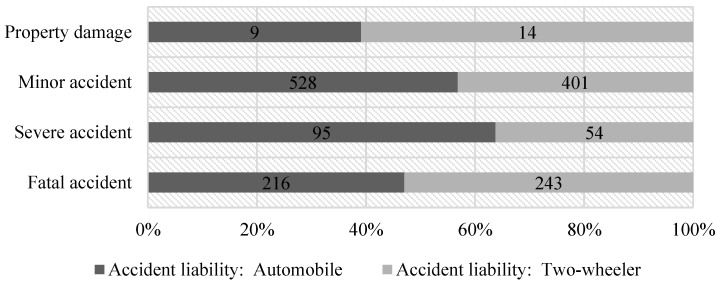
Proportion of two types of accidents in different levels.

Specially, traffic accidents were divided into two categories, namely, traffic accidents in which the automobile was the primary responsible party and in which the two-wheeler was the primary responsible party. There are 848 accidents in which the automobile was the primary responsible party and 712 accidents in which the two-wheeler was the primary responsible party. The differences in the proportion of these two types of accidents in different levels prove the necessity of the classification, as shown in Figure 1.

### 3.3. Sample Set Quantization and Data Discretization

The influencing factors of accident severity were divided into the driving behavior and driver characteristics for automobiles and two-wheeled vehicles, road characteristics, peak time, and environmental characteristics [19,20,21]. This paper collated the data of automobile to two-wheeler traffic accidents in Guilin, including 24 indicators from four aspects.

According to the requirements of the BN model, the classification and coding of each node should be quantified and discretized [22,23,24]. Quantization is the assignment of a value to each property of an item. Discretization is the mapping of the assignment of a continuous variable to several uncorrelated intervals [25]. The results of quantization and discretization of the sample set data are shown in Table 2.

## 4. Methodology

Firstly, the key factors for the construction of BN were selected by carrying out the Kendall rank correlation analysis on the influencing factors of automobile to two-wheeler traffic accidents; secondly, the structure learning and parameter learning methods based on the CH (Calinski–Harabasz) score were applied to construct the BN model; finally, the node probability estimated by Bayesian estimation (BE) was compared with the actual probability to verify the validity of the model.

### 4.1. Kendall Rank Correlation Coefficient Analysis Method for Determining the Key Factors

The purpose of establishing the correlation analysis method is to screen and rank the key factors affecting the severity of automobile to two-wheeler traffic accidents. Meanwhile, it is necessary to use the linear correlation degree among variables in the process of establishing the BN model, so the Kendall rank correlation coefficient is more suitable as the measurement standard for the correlation analysis.

**Figure 2:**
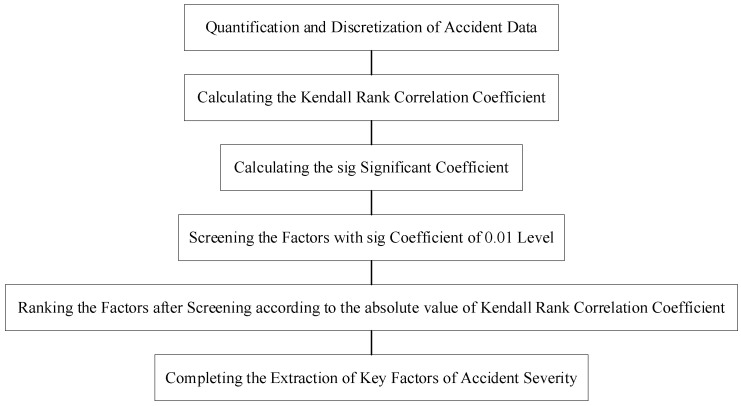
Key factor identification process based on the Kendall rank correlation analysis.

As shown in Figure 2, the traffic accident datasets for the automobile and two-wheeler vehicle attributed to the primary responsibility are input to calculate the Kendall rank correlation coefficients between the influential factors and the severity, as shown in Equation (1). Then, the significance analysis of the correlation coefficients is achieved by constructing a t-statistic, and the significance coefficients are calculated as Equation (2). Finally, the influence factors with significant correlation were selected as the key factors affecting the severity of the accident.

(1) $$r=\frac{\sum_{i<j}\mathrm{sgn}(x_{i}-x_{j})\mathrm{sgn}(y_{i}-y_{j})}{\sqrt{\left(T_{0}-T_{1})(T_{0}-T_{2}\right)}}$$

(2) $$T=\frac{r\sqrt{n-2}}{\sqrt{1-r}}$$

where $\mathrm{sgn}(z)=\{\begin{array}{c} 1\\ 0\\ -1 \end{array}\begin{array}{c} \left(z>0\right)\\ \left(z=0\right)\\ \left(z<0\right) \end{array}$; $T_{0}=\frac{n(n-2)}{2}$, n represents the total number of observations; $T_{1}=\frac{\sum t_{i}(t_{i}-1)}{2}$, $T_{2}=\frac{\sum u_{i}(u_{i}-1)}{2}$, $t_{i}$ and $u_{i}$ represent the number of ith type in x and y, respectively.

### 4.2. Bayesian Network Accident Severity Analysis Modeling

According to Bayes theorem and the chain rule of conditional probability, supposing the random variable corresponding to node k is $X={\left(X_{k}\right)}_{k\in K}$, then the joint probability of node k is as defined in Equation (3).

(3) $$P(K)=\prod_{k\in K}P(X_{k}|X_{1},X_{2},X_{3},\ldots ,X_{k-1})=\prod_{k\in K}P(X_{k}|X_{node(k)})$$

where $X_{node(k)}$ represents the parent node of node k. With the probability value of the input variable (evidence variable), the probability distribution of the output variable can be calculated according to the existing BN structure and the conditional probability table (CPT). The logical relationship between nodes in the network model can be expressed as the propagation of conditional probability, which makes the network reasoning analysis possible.

#### 4.2.1. Learning Bayesian Network Structure Based on CH Scoring Method and K2 Algorithm

The purpose of structure learning is to find a directed acyclic graph (DAG) from the sample data that can best represent the relationship between the influencing factors. The principle is to learn network structure based on corresponding scoring criteria and search strategies. The structural learning of the BN accident severity analysis model was established by using the CH scoring standard and the K2 algorithm.

The learning of BN structure can be expressed as defined in Equation (4):

(4) $$\left\{ \begin{array}{c} \max f(N,D)\\ s.t.N\in \Phi ,N|=C \end{array}\right.$$

where Φ represents the BN structure, f(N,D) represents the score value of the network structure, and N|=C represents that the node N in the network structure satisfies the restriction of the constraint C. The BN structure after structural learning is as defined in Equation (5).

(5) $$N^{*}=\arg\max_{N}P(N|D)=\arg\max_{N}\frac{P(D|N)P(N)}{P(D)}$$

where P(N|D) represents the posterior probability of network structure N, and P(N) represents the prior probability of network structure *N*.

The network structure iteration steps are as follows (K2 algorithm):

*Step 1: Select the impact factor as the BN node.*

*Step 2: Initialize the network structure. Enter the node order* $order=\{x_{1},x_{2},x_{3},\ldots ,x_{n}\}$ *, the node with the lower node order cannot be used as the parent node of the node with the upper order.*

*Step 3: Calculate the score* $V=CH(x_{k},x_{node(k)}|D)$ *under the network structure according to the scoring function and update the parent node according to Step 4.*

*Step 4: Judge the number of parent nodes, if the number of parent nodes is less than 3, continue the search, and give priority to nodes without parent nodes in the search process. If* $\left\{V_{new}=CH(x_{k},x_{node(k)}\cup x_{l}|D)\}>\{V_{old}=CH(x_{k},x_{node(k)}|D)\right\}$ *, then node* $x_{l}$ *is regarded as the new parent node of* $x_{k}$ *. Repeat the above process until the search is completed.*

*Step 5: Connect the node and the parent node to generate a BN directed acyclic graph*.

#### 4.2.2. Learning Bayesian Network Parameters Based on Bayesian Estimation

The purpose of parameter learning is to use sample data to quantify the interdependence between BN nodes. The parameter learning method used in this paper is BE, which can combine prior knowledge and training sample data sets to improve the accuracy of the model. The specific mechanism is as follows:

Assuming that the prior probability of the network parameters is P(λ), search for the parameter with the largest posterior probability through the training sample data set $D=\{x_{1},x_{2},x_{3},\ldots ,x_{n}\}$. The formula for calculating the posterior probability is as defined in Equation (6).

(6) $$P(\lambda |D)=\frac{P(D|\lambda )}{P(D)}P(\lambda )$$

According to the law of total probability P(D)=∫P(D|λ)P(λ)dλ, when the samples are independent from each other, $P(D|\lambda )=\prod_{i=1}^{n}P(x_{i}|\lambda )$. Then the following is deduced:

(7) $$P(\lambda |D)=\frac{\left(\prod_{i=1}^{n}P(x_{i}|\lambda ))P(\lambda \right)}{\int (\prod_{i=1}^{n}P(x_{i}|\lambda ))P(\lambda )d\lambda }$$

(8) $$\hat{\lambda }=E(\lambda |D)=\int \lambda P(\lambda |D)d\lambda$$

Due to the conjugate nature of the Dirichlet distribution, the computational complexity of the BN model can be greatly reduced.

After the BN structure is determined through structural learning, the probability relationship between nodes can be described by conditional probability. Assuming that the prior probability distribution of each node variable satisfies the Dirichlet distribution, the BN parameter learning method is used to learn the conditional probability of each node under different contribution factors. Then, the BN joint tree engine combination is used to realize the factor combination sorting. Finally, the effectiveness of the model is tested by comparing the learning results with the real situation.

## 5. Results

### 5.1. Identification Results of Key Factors of Two Types of Traffic Accidents

According to the principle of significance testing, a factor with a coefficient of sig. value less than 0.01 is a critical factor with significant correlation Therefore, the factors affecting traffic accidents were screened according to the sig. value, it can be concluded that 10 critical factors were related to the severity of the traffic accidents attributed to the automobile as the primary responsible party. In the traffic accidents that attributed the two-wheeler as the primary responsible party, 12 key factors were related to the severity of the accident.

**Figure 3:**
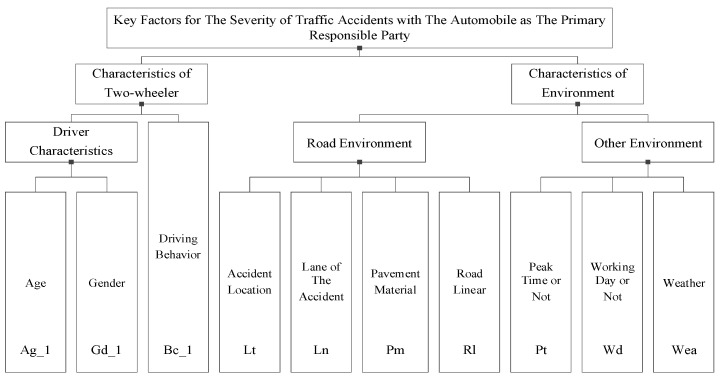
Set of key factors in traffic accidents with the automobile as the primary responsible party.

**Figure 4:**
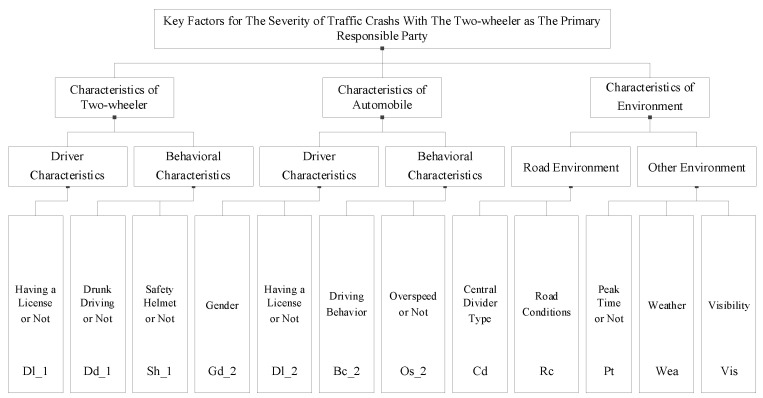
Set of key factors in traffic accidents with the two-wheeler as the primary responsible party.

The set of key influencing factors of traffic accidents with the automobile as the primary responsible party and the two-wheeler as the primary responsible party are shown in Figure 3 and Figure 4, respectively.

Then, sequential sequences are generated based on the absolute values of the Kendall correlation coefficients (shown in Table 2) to satisfy the requirements of Bayesian network structure learning. The sequencing results are shown in Table 3.

### 5.2. Bayesian Network Modeling Results of the Severity of Two Types of Traffic Accidents

(*i*). *Bayesian Network Structure Learning* *Results*

**Figure 5:**
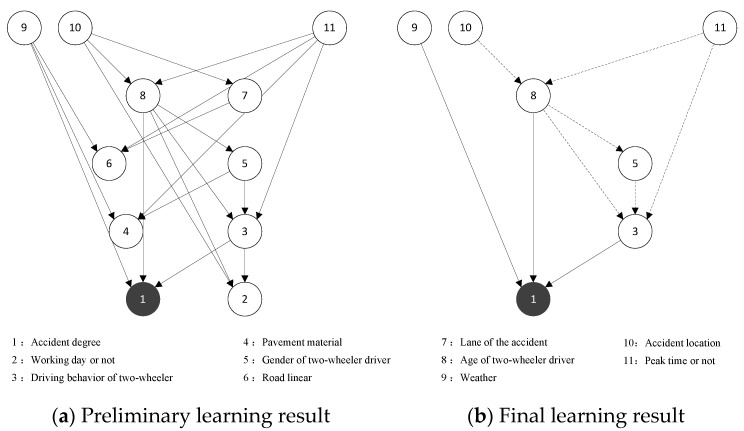
Automobile as the primary responsible party traffic accident BN structure learning results. Node: The solid and dashed lines represent the direct and indirect impact, respectively.

**Figure 6:**
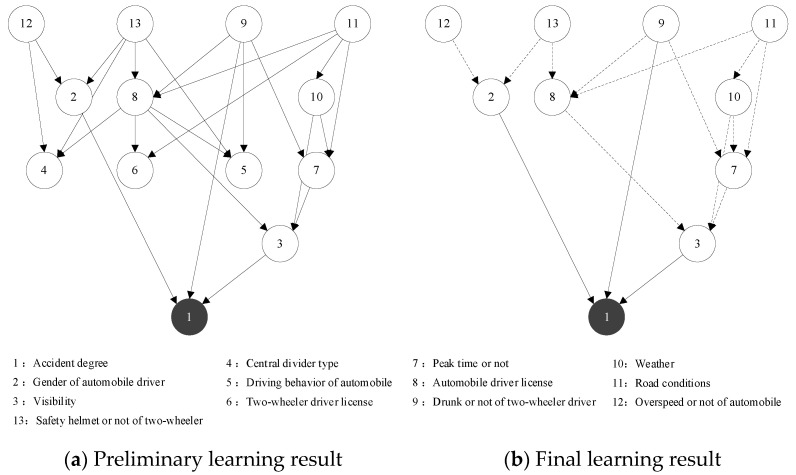
Two-wheeler as the primary responsible party traffic accident BN structure learning results. Node: The solid and dashed lines represent the direct and indirect impact, respectively.

Based on the results of the correlation analysis, the key factors of the traffic accident for automobiles and two-wheelers as the primary responsible parties were obtained as the network nodes in the BN. The dependent variable was the severity of the traffic accident (Ad), and the others were the independent variables, including weather (Wea), peak time or not (Pt), etc. For the traffic accident where the automobile is the primary responsible party, this paper selected 11 nodes in total, and the preliminary learning results of the BN structure are shown in Figure 5a. For traffic accidents with the two-wheeler as the primary responsible party, a total of 13 nodes were selected and the preliminary learning results of the BN structure are shown in Figure 6a.

As shown in Figure 5, nodes 3 (Bc_1), 8 (Ag_1), and 9 (Wea) directly affect the result variable node 1 (Ad), but nodes 2 (Wd), 4 (Pm), 6 (Rl), and 7 (Ln) fail to be associated with the severity of the traffic accident node 1. Therefore, the four node variables of working day or not, pavement material, road linear, and lane of the accident were deleted from the first traffic accident network. The nodes that have a direct impact on the severity of traffic accident variables are distinguished from the other indirect impact nodes (the solid and dashed lines represent the direct and indirect impact, respectively). The final result of BN structure learning of the traffic accident with the automobile as the primary responsible party was obtained as shown in Figure 5b. Similarly, by deleting nodes 4 (Cd), 5 (Bc_2), and 6 (Dl_1) from the second traffic accident network and distinguished between directly affecting nodes and indirectly affecting nodes, then the final result of the BN structure learning of the traffic accident with the two-wheeler as the primary responsible party was obtained as shown in Figure 6b.

As shown in Figure 5b, the factors affecting the severity of traffic accident for automobiles as the primary responsible party are as follows:

1. The direct influential factors: driving behavior of the two-wheeler, age of two-wheeler driver, and weather.
2. The longest influential factor sequences: peak time or not → age of two-wheeler driver → gender of two-wheeler driver → driving behavior of two-wheeler → accident degree; accident location → age of two-wheeler driver → gender of two-wheeler driver → driving behavior of two-wheeler → accident degree.

As shown in Figure 6b, the factors affecting the severity of the traffic accident attributed to the two-wheeler as the primary responsible party are as follows:

1. The direct influential factors: gender of the automobile driver, visibility, and drunk or not of the two-wheeler driver.
2. The longest influential factor sequences: road conditions → weather → peak time or not → visibility → accident degree.

(*ii*). *Bayesian Network Parameter Learning Results*

Assuming that the prior probability distribution of each node in the BN of traffic accident severity obeys the Dirichlet distribution, the conditional probability of the factors affecting the severity of the traffic accident was calculated. The results are shown in Table 4 and Table 5.

For the severity of traffic accidents in which the automobile and two-wheeler are the primary responsible parties, respectively, the influential degrees of different factors are very different. Taking the fatal accidents and severe accidents as examples, the influential degree of each critical factor was calculated for ranking and sorting the key nodes (taking the weather as an example in Table 4, the probability of fatal and severe accidents is the highest when it is rainy, 0.4263 + 0.0674 = 0.4937, and the probability of fatal and severe accidents is the lowest when it is cloudy, 0.1043 + 0.0562 = 0.1605, finally the weather’s influence degree is calculated as 0.4937 − 0.1605 = 0.3332). As shown in Table 4 and Table 5, when the automobile is the primary responsible party, the fatal and severe accidents were considered together, the age of the two-wheeler and the driving behavior of the two-wheeler are the most influential factors. Similarly, when the two-wheeler is the primary responsible party, the most influential factor is visibility, followed by gender of the automobile and drunk driving or not of the two-wheeler.

(*iii*). *Combination Ranking Results of Traffic Accident Severity Factors*

The severity of traffic accidents is affected by multiple factors. In addition to analyzing the impact of a single factor on traffic accidents, the probability distribution of the severity of traffic accidents under a combined influence of multiple factors should also be identified. The probability distributions of fatal accidents were applied as the sorting standard to analyze and sort the multiple influencing factors of the severity of traffic accidents with automobiles and two-wheelers as the primary responsible parties. The sorting results are shown in Table 6.

Studying the multi-factor combination sequence can help to better understand the causes of accidents and provide the effective measures for Departments of Transportation. Regarding the accidents where the automobile is the primary responsible party, during the peak period, traffic accidents with driving of the two-wheeler by elderly drivers are the most likely to cause death. At the same time, minors are also prone to fatal accidents when they drive two-wheeled vehicles straight ahead. Therefore, the studied counter measures should be focused on safeguarding minors and the elderly from driving two-wheeled vehicles to minimize serious traffic accidents. For example, optimizing the road channelization layout to separate two-wheeled vehicles from automobiles, in particular, safer isolation facilities should be installed on roads with a large number of minors and elderly people; secondly, signal lights and warnings should be installed for zebra crossings with a large number of people to provide a safer environment for pedestrians to cross the road, etc. Regarding the accidents where the two-wheeler is the primary responsible party, the top factors of all the sequences which are also very likely to cause death include visibility and two-wheeled vehicle drivers with drunk driving. Therefore, we should focus on researching strategies to ensure driving safety when visibility is affected and strengthen the enforcement and punishment of drunk driving to improve drivers’ awareness of safe driving.

(*iv*). *Model Validity Test Results*

According to the comparison between the prediction results of the BN model and the statistical calculation results of the original testing datasets, the learning error of the BN model can be obtained to evaluate the accuracy of the model. The dependent variable is the severity of the traffic accident, so the model learning result of the node of the severity of the accident was selected for validity testing. The testing results are shown in Table 7 and Table 8.

As shown in Table 7 and Table 8, for the prediction of the traffic accident severity with two-wheelers as the primary responsible party, the maximum absolute error is 0.1303, the overall average error is 0.0170, and the average error after removing extreme scenes with very few samples is 0.0091. For the prediction of the traffic accident severity with automobiles as the primary responsible party, the maximum absolute error is as high as 0.2493. That is because the small number of traffic accidents of this type leads to poor model prediction results, but the overall average error is only 0.0520. After removing extreme scenes with very few samples, the average error is 0.0283, which meets the prediction accuracy. Therefore, the proposed BN model can be used to analyze the severity of automobile to two-wheeler traffic accidents.

### 5.3. Comparison with Unclassified Bayesian Network Model

**Figure 7:**
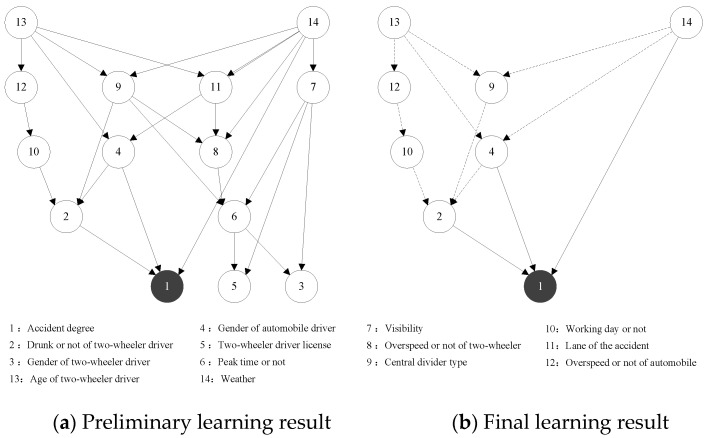
Complete automobile to two-wheeler traffic accident BN structure learning results. Note: The solid and dashed lines represent the direct and indirect impact, respectively.

To better explore the heterogeneity of accidents with different accident responsible parties, the Bayesian network modeling of the accident sample dataset without classification is performed in this section and compared with the above results. The sequence of key influencing factors is filtered and ranked by calculating the Kendall correlation coefficients as shown in Table 9, and the Bayesian network structure of the complete dataset is subsequently trained as shown in Figure 7.

As shown in Figure 7b, the factors affecting the severity of traffic accidents are as follows:

1. The direct influential factors: drunk or not of two-wheeler driver, gender of automobile driver, and weather.
2. The longest influential factor sequences: age of two-wheeler driver → speeding or not of automobile driver → working day or not → drunk or not of two-wheeler driver → accident degree.

Comparing the direct influential factors of the complete dataset with the two previous traffic accident datasets, we found that the three sets of direct influential factors are not exactly the same, but the three direct influencing factors of the complete dataset (drunk or not of two-wheeler driver, gender of automobile driver, and weather) are all included in the other two sets of direct influences, at the same time, the remaining indirect influential factors almost have the same performance. Together, this indicates that the analysis results classified according to the responsible party have both commonality and specialty with the results of the complete dataset. Therefore, the heterogeneity of automobile and two-wheeler traffic accidents can be successfully explored by the method proposed in this paper.

## 6. Discussion and Conclusions

This paper used the BN model to study automobile–two-wheeler traffic accidents. Taking into account the difference in the division of the primary responsibility, the automobile–two-wheeler traffic accidents were divided into the automobile and the two-wheeler as the primary responsible party. Based on the traffic accident dataset of Guilin City, Guangxi Province, the paper separately studied the key factors affecting the severity of the accident in the traffic accidents of different accident responsible parties, explored the internal connections between the critical factors, and obtained the following conclusions.

1. The direct factors that affect the severity of traffic accidents in which the automobile is the primary responsible party are the driving behavior of two-wheelers, the age of two-wheeler drivers, and weather. For traffic accidents which the two-wheeler is the primary responsible party, the direct factors are the gender of the automobile driver, visibility, and drunk driving or not of the two-wheeler driver. Under the influence of a single factor, in the traffic accident where the automobile is the primary responsible party, the biggest factor that causes death is weather. The probability of death under different weather conditions fluctuates by 32.2%. The age of the two-wheeler driver is the factor that affects the occurrence of death and serious injuries the most. For traffic accidents in which two-wheelers are the primary responsible party, visibility is the biggest factor leading to death or serious injury. Under the influence of visibility, the probability of death event fluctuates by 27.23%, and the probability of combined death and serious injury accidents fluctuates by 22.37%.
2. The key influencing factors of traffic accidents for different accident responsible parties are very different. Only whether peak time and weather factors are the common key factors affecting the severity of traffic accidents for both the automobile and the two-wheeler as the main responsible party. When the automobile is mainly responsible for traffic accidents, the severity of the accident does not depend on the physical characteristics of the vehicle driver, but more depends on the road environment, road conditions, natural environment, and the physical capacity of the two wheeled vehicle driver. Therefore, the critical factors affecting the traffic accident severity are concentrated on the characteristics of the driving environment and the two-wheeler driver. However, for traffic accidents in which a two-wheeler is the primary responsible party, the severity of the accident often depends on the driving ability of the two-wheeler driver and the stress behavior of the vehicle driver when the vehicle is pre-crashed. In addition to driving environmental factors, automobile driver characteristics, automobile driving behavior characteristics, self-driving behavior, and self-driving ability would also affect the severity of the accident.
3. Through the combination analysis of key factors, the probability distribution of accident severity under different factor scenarios can be inferred. For traffic accidents where the automobile is the primary responsible party, when the combined sequence of the factors status is {Off-Peak Period → Driver of Two-Wheeler: The elderly → Driving Behavior of Two-Wheeler: Parking}, the probability of death is the largest, as high as 67.9%. Among the traffic accidents in which the two-wheeler is the primary responsible party, the accidents under the combination of factors {Drunk Driving Two-Wheeler → Having a License of Automobile → Visibility: 50 m~100 m} are the most likely to cause death and the probability is 63.59%. The reasonable analysis of this series of status sequences can provide sufficient theoretical support for the active prevention of serious traffic accidents. By applying state sequence to medical aid systems and traffic law enforcement systems, a data-driven and scenario-driven intelligent coordination system can be constructed. Taking the high-risk sequence {Drunk Driving Two-Wheeler → Having a License of Automobile → Visibility: 50 m~100 m} as an example, if the current visibility is 50 m~100 m, the traffic safety administration department should focus on the investigation of drunk driving behavior of two-wheeled vehicles and the inspection of automobile driving licenses, so as to avoid serious traffic accidents as much as possible.

In addition, through the analysis of the results, there is a conclusion of discrepancy with subjective recognition: the factors that affect the severity of traffic accidents in which the automobile is the primary responsible party are mainly related to the characteristics of the environment and the characteristics of the two-wheeler, and do not include the characteristics of the automobile. Additionally, there are two main reasons: On the one hand, regardless of the type of accident, the more serious casualties tend to be on the weaker side (the two-wheeled vehicle), so the characteristics of two-wheeler itself must be more influential. After all, older drivers of two-wheelers are usually more severely injured than adult drivers when they are subjected to the same level of collision. On the other hand, driving violations for automobiles in China are more stringent both in terms of education and punishment than two-wheeled vehicles, and it leads to the fact that the automobile drivers with driving violations usually conform to survivorship bias, i.e., drivers who choose to drive automobiles illegally are generally better than the others. Therefore, the mining of influencing factors from the data perspective might be different from the subjective recognition.

A few limitations exist in this paper. On the one hand, scholars have increasingly focused on the study of the unobserved heterogeneity [26,27,28,29]; in this paper, the study is conducted only from the perspective of the responsible party of the accident, ignoring the temporal heterogeneity and spatial heterogeneity of automobile and two-wheeler accidents. Therefore, a more comprehensive study of the heterogeneity of data samples will be conducted in the next work. On the other hand, this paper only undertook a theoretical analysis of the traffic safety strategy and cannot evaluate the effectiveness of the strategy. Therefore, the next work will simulate the road environment before and after the use of the safety strategy, and design indicators to measure the effectiveness of the traffic safety strategy.

## Figures and Tables

**Table 1:** Raw data example.

Accident Number	Accident Degree	Accident Characteristics	Accident Causes	Accident Liability	Automobile Information	Two-Wheeler Information
45030272 01500013	1	2015/3/17 23:20 Working days; the peak hour; 100 m north of Jiashan Road	Driving an automobile after drinking alcohol	1	Drunk driving; not exceeding the speed limit	Not exceeding the speed limit; 30 years old

Note: Accident degree: 1 = fatal accident; 2 = severe accident; 3 = minor accident; 4 = property damage. Accident liability: 1 = automobile as the primary responsible party; 2 = two-wheeler as the primary responsible party.

**Table 2:** Influencing factors and data discretization of automobile to two-wheeler traffic accidents.

Variable Classification	Variables	Symbol	Variables Assignment				Kendall Correlation	
Dependent	Accident degree	Ad	1 = Fatal accident		2 = Severe accident		Two-wheeler	Auto mobile
			3 = Minor accident		4 = Property damage			
Driving Behavior and Driver Characteristics of Two-Wheeler	Behavioral characteristics	Bc_1	1 = Go Straight		2 = Turn Left		-	−0.138
			3 = Turn Right		4 = Parking			
			5 = Cross Street					
	Gender	Gd_1	1 = Male		2 = Female		-	0.120
	Age	Ag_1	1 = Minor		2 = Youth (18–35)		-	−0.089
			3 = Wrinkly (36–55)		4 = The elderly (>55)			
	Having a license or not	Dl_1	1 = Yes		2 = No		0.102	-
	Drunk driving or not	Dd_1	1 = Yes		2 = No		0.090	-
	Speeding or not	Os_1	1 = Yes		2 = No		-	-
	Safety helmet or not	Sh_1	1 = Yes		2 = No		0.073	-
Driving Behavior and Driver Characteristics of Automobile	Behavioral characteristics	Bc_2	1 = Go Straight		2 = Turn Left		0.132	-
			3 = Turn Right		4 = Parking			
			5 = Cross Street					
	Gender	Gd_2	1 = Man		2 = Woman		0.184	-
	Age	Ag_2	1 = Minor		2 = Youth (18–35)		-	-
			3 = Wrinkly (36–55)		4 = The elderly (>55)			
	Having a license or not	Dl_2	1 = Yes		2 = No		0.099	-
	Drunk driving or not	Dd_2	1 = Yes		2 = No		-	-
	Speeding or not	Os_2	1 = Yes		2 = No		0.079	-
Characteristics of Road Type	Accident location	Lt	1 = Segment				-	−0.076
			2 = Intersection with Control					
			3 = Intersection without Control					
	Lane of the accident	Ln	1 = Vehicular Lane		2 = Other Lane		-	0.093
	Central divider type	Cd	1 = No Barrier		2 = Barrier		−0.132	-
			3 = Green Belt					
	Pavement material	Pm	1 = Pitch		2 = Cement		-	0.132
	Road conditions	Rc	1 = Intact		2 = Broken		0.086	-
	Road linear	Rl	1 = Linear		2 = Non-Linear		-	−0.098
Characteristics of Peak Time and Environment	Working day or not	Wd	1 = Yes		2 = No		-	−0.142
	Peak time or not	Pt	1 = Off-peak time		2 = Peak time		0.102	0.067
	Lighting condition	Lc	1 = Daytime		2 = Nighttime		-	-
	Weather	Wea	1 = Sunny	2 = Cloudy		3 = Rainy	−0.087	0.079
	Visibility	Vis	1 = <50 m		2 = 50 m~100 m		0.144	-
			3 = 100 m~200 m		4 = >200 m			

Note: “-” represents that the corresponding variable has no significant correlation. In the Kendall correlation, the first column corresponds to the traffic accident in which the two-wheeler is the primary responsible party; the second column corresponds to the traffic accident in which automobile is the primary responsible party. All variables are extracted from the raw data.

**Table 3:** Sequencing results based on the absolute values of the Kendall correlation coefficients.

Accident Type	Sequential Sequence of the Kendall Correlation Coefficients											
Automobile as the Primary Responsible Party	1	Wd	2	Bc_1	3	Pm	4	Gd_1	5	Rl	6	Ln
	7	Ag_1	8	Wea	9	Lt	10	Pt				
Two-Wheeler as the Primary Responsible Party	1	Gd_2	2	Vis	3	Bc_2	4	Cd	5	Pt	6	Dl_1
	7	Dl_2	8	Dd_1	9	Wea	10	Rc	11	Os_2	12	Sh_1

**Table 4:** Conditional probability of influencing factors of traffic accidents with the automobile as the primary responsible party.

Influencing Factors Variable		Probability of Different Accident Levels				Influence Degree	Ranking
		Fatal Accident	Severe Accident	Minor Accident	Property Damage		
Driving Behavior of Two-Wheeler	Go Straight	0.2080	0.1188	0.6640	0.0093	0.3675	2
	Turn Left	0.3118	0.2866	0.3873	0.0143		
	Turn Right	0.4145	0.1344	0.3166	0.1344		
	Parking	0.1675	0.0634	0.3507	0.4185		
	Across Street	0.4033	0.1034	0.4783	0.0150		
Gender of Two-Wheeler	Man	0.2523	0.1342	0.5946	0.0189	0.0711	4
	Woman	0.2025	0.1129	0.6373	0.0474		
Age of Two-Wheeler	Minor	0.4268	0.3889	0.1067	0.0776	0.5108	1
	Youth (18–35)	0.1946	0.1468	0.6334	0.0252		
	Wrinkly (36–55)	0.1750	0.1299	0.6628	0.0322		
	The elderly (>55)	0.4162	0.0586	0.5210	0.0042		
Accident Location	Segment	0.2317	0.1206	0.6251	0.0226	0.0309	6
	Intersection with Ctrl	0.2647	0.1185	0.5942	0.0226		
	Intersection no Ctrl	0.2416	0.1371	0.5941	0.0272		
Peak Time or Not	Off-Peak Period	0.2513	0.1283	0.5967	0.0236	0.0336	5
	Peak Period	0.2133	0.1327	0.6247	0.0294		
Weather	Sunny	0.2447	0.1789	0.5451	0.0314	0.3332	3
	Cloudy	0.1043	0.0562	0.8299	0.0096		
	Rainy	0.4263	0.0674	0.4798	0.0265		

**Table 5:** Conditional probability of influencing factors of traffic accidents with the two-wheeler as the primary responsible party.

Influencing Factors Variable		Probability of Different Accident Levels				Influence Degree	Ranking
		Fatal Accident	Severe Accident	Minor Accident	Property Damage		
Drunk Driving or Not of Two-Wheeler	Yes	0.4678	0.0899	0.3967	0.0456	0.1579	3
	No	0.2996	0.1002	0.5904	0.0098		
Safety Helmet or Not of Two-Wheeler	Yes	0.3452	0.1012	0.5382	0.0154	0.0121	7
	No	0.3367	0.0976	0.5476	0.0181		
Gender of Automobile	Man	0.3486	0.1021	0.5359	0.0134	0.2145	2
	Woman	0.1929	0.0433	0.6879	0.0759		
Having a License or Not of Automobile	Yes	0.3594	0.097	0.5223	0.0213	0.0283	6
	No	0.3299	0.0982	0.5553	0.0166		
Speeding or Not of Automobile	Yes	0.3367	0.0973	0.5487	0.0173	0.0014	8
	No	0.3375	0.0979	0.5467	0.0179		
Road Conditions	Intact	0.3373	0.0981	0.5467	0.0179	0.0007	9
	Broken	0.3516	0.0831	0.5471	0.0182		
Peak Time or Not	Off-Peak Period	0.3504	0.0961	0.5341	0.0194	0.0590	5
	Peak Period	0.2821	0.1054	0.6013	0.0112		
Weather	Sunny	0.3178	0.1062	0.5589	0.0171	0.1091	4
	Cloudy	0.2922	0.1058	0.5864	0.0156		
	Rainy	0.4434	0.0637	0.4705	0.0224		
Visibility	<50 m	0.5147	0.0197	0.4459	0.0197	0.2237	1
	50 m~100 m	0.5169	0.0582	0.3928	0.0321		
	100 m~200 m	0.2446	0.1068	0.6344	0.0142		
	>200 m	0.2629	0.1262	0.5980	0.0129		

**Table 6:** Combination ranking of multi-influencing factors in fatal accidents.

Accident Type	Multi-factor Combination Sequence	Fatal Accident	
		Probability	Ranking
Automobile as the Primary Responsible Party	Off-Peak Period → Driver of Two-Wheeler: The elderly → Driving Behavior of Two-Wheeler: Parking	0.6790	1
	Intersection without Control → Driver of Two-Wheeler: The elderly → Driving Behavior of Two-Wheeler: Across Street	0.6672	2
	Driver of Two-Wheeler: The elderly → Driving Behavior of Two-Wheeler: Across Street	0.6672	3
	Intersection without Control → Driver of Two-Wheeler: The elderly → Driving Behavior of Two-Wheeler: Turn Left	0.5587	4
	Off-Peak Period → Driver of Two-Wheeler: Minor →Driving Behavior of Two-Wheeler: Go Straight	0.4790	5
Two-Wheeler as the Primary Responsible Party	Drunk Driving Two-Wheeler → Having a License of Automobile → Visibility: 50 m~100 m	0.6359	1
	Drunk Driving Two-Wheeler → Off-Peak Period → Visibility: 50 m~100 m	0.6336	2
	Safety Helmet of Two-Wheeler → Having no License of Automobile → Visibility: <50 m	0.5967	3
	Road Broken → Off-Peak Period → Visibility: <50 m	0.5776	4
	Road Broken → Rainy → Visibility: <50 m	0.5313	5

**Table 7:** Validity test results of the Bayesian network model of traffic accidents with the automobile as the primary responsible party.

Direct Influencing Factors		Result Variable: Accident Degree											
		Fatal Accident			Severe Accident			Minor Accident			Property Damage		
		Bayes	Real Value	Absolute Error	Bayes	Real Value	Absolute Error	Bayes	Real Value	Absolute Error	Bayes	Real Value	Absolute Error
Bc_1	1	0.208	0.2128	0.0048	0.1188	0.1135	0.0053	0.664	0.6667	0.0027	0.0093	0.0071	0.0022
	2	0.3118	0.2500	0.0618	0.2866	0.1875	0.0991	0.3873	0.5625	0.1752	0.0143	0.0000	0.0143
	3	0.4145	0.5000	0.0855	0.1344	0.0000	0.1344	0.3166	0.5000	0.1834	0.1344	0.0000	0.1344
	4	0.1675	0.2000	0.0325	0.0634	0.0000	0.0634	0.3507	0.6000	0.2493	0.4185	0.2000	0.2185
	5	0.4033	0.4762	0.0729	0.1034	0.0952	0.0082	0.4783	0.4286	0.0497	0.0150	0.0000	0.0150
Ag_1	1	0.4268	0.5000	0.0732	0.3889	0.3333	0.0556	0.1067	0.1667	0.0600	0.0776	0.0000	0.0776
	2	0.1946	0.1884	0.0062	0.1468	0.1159	0.0309	0.6334	0.6812	0.0478	0.0252	0.0145	0.0107
	3	0.1750	0.1781	0.0031	0.1299	0.1233	0.0066	0.6628	0.6849	0.0221	0.0322	0.0137	0.0185
	4	0.4162	0.4615	0.0453	0.0586	0.0513	0.0073	0.5210	0.4872	0.0338	0.0042	0.0000	0.0042
Wea	1	0.2447	0.2752	0.0305	0.1789	0.1651	0.0138	0.5451	0.5413	0.0038	0.0314	0.0183	0.0131
	2	0.1043	0.0870	0.0173	0.0562	0.0435	0.0127	0.8299	0.8696	0.0397	0.0096	0.0000	0.0096
	3	0.4263	0.4063	0.0200	0.0674	0.0313	0.0361	0.4798	0.5625	0.0827	0.0265	0.0000	0.0265
Average Error:			0.0520			Average Error after Removing Extreme Scenes:					0.0283		

**Table 8:** Validity test results of the Bayesian network model of traffic accidents with the two-wheeler as the primary responsible party.

Direct Influencing Factors		Result Variable: Accident Degree											
		Fatal Accident			Severe Accident			Minor Accident			Property Damage		
		Bayes	Real Value	Absolute Error	Bayes	Real Value	Absolute Error	Bayes	Real Value	Absolute Error	Bayes	Real Value	Absolute Error
Dd_1	1	0.4678	0.4444	0.0234	0.0899	0.0833	0.0066	0.3967	0.4167	0.0200	0.0456	0.0556	0.0100
	2	0.2553	0.3040	0.0044	0.1002	0.0960	0.0042	0.5904	0.5920	0.0016	0.0098	0.0080	0.0018
Gd_2	1	0.3002	0.3533	0.0047	0.1021	0.1000	0.0021	0.5359	0.5333	0.0026	0.0134	0.0134	0.0000
	2	0.2651	0.0909	0.1020	0.0433	0.0000	0.0433	0.6879	0.8182	0.1303	0.0759	0.0909	0.0150
Vis	1	0.2924	0.5294	0.0147	0.0197	0.0000	0.0197	0.4459	0.4706	0.0247	0.0197	0.0000	0.0197
	2	0.2472	0.5000	0.0169	0.0582	0.0588	0.0006	0.3928	0.4118	0.0190	0.0321	0.0294	0.0027
	3	0.1723	0.2414	0.0032	0.1068	0.1034	0.0034	0.6344	0.6207	0.0137	0.0142	0.0345	0.0203
	4	0.3449	0.2593	0.0036	0.1262	0.1235	0.0027	0.5980	0.6049	0.0069	0.0129	0.0123	0.0006
Average Error:			0.0170			Average Error after Removing Extreme Scenes:				0.0091			

**Table 9:** Sequencing results based on the absolute values of the Kendall correlation coefficients.

Accident Type	Sequential Sequence of the Kendall Correlation Coefficients						
Complete Automobile to Two-Wheeler Traffic Accident	1	2	3	4	5	6	7
	Dd_1	Gd_1	Gd_2	Dl_1	Pt	Vis	Os_1
	8	9	10	11	12	13	
	Cd	Wd	Ln	Os_2	Ag_1	Wea	

## Data Availability

All data used in this study were obtained from the Department of Transportation Guilin City, Guangxi Province, China. and described in Section 3.
